# Alterations in Cerebral Intrinsic Activity in First-Episode, Drug-Naive Patients With Major Depressive Disorder

**DOI:** 10.7759/cureus.83737

**Published:** 2025-05-08

**Authors:** Ali Tariq, Asma Ul Hussain, Youmnah Ali, Hafiza Sidra, Hamail Hashmi, Bushra Imtiaz, Hamza Shahab, FNU Partab, FNU Shweta

**Affiliations:** 1 General Internal Medicine, Beaumont Hospital, Dublin, IRL; 2 Medicine, Agha Khan Health Facility, Rawalpindi, PAK; 3 Medicine, Noor Medical Center, Rawalpindi, PAK; 4 Internal Medicine, University of Health Sciences, Lahore, PAK; 5 Medicine, Khawaja Muhammad Safdar Medical College, Sialkot, PAK; 6 Medicine, Qasmi Eye &amp; Gynae Hospital, Bhimber, PAK; 7 Internal Medicine, Idrees Hospital, Sialkot, PAK; 8 Internal Medicine, Chandka Medical College, Larkana, PAK; 9 Epidemiology and Biostatistics, Drexel University, Philadelphia, USA

**Keywords:** depressive disorder, drug devlopment, major trauma, mri images, radio frequency

## Abstract

Background: The amplitude of low-frequency fluctuation (ALFF) obtained by resting-state functional magnetic resonance imaging (rs-fMRI) has been widely used to measure cerebral intrinsic neural activity in major depressive disorder (MDD). The primary objective is to investigate alterations in cerebral intrinsic activity, as measured by ALFF, in first-episode, drug-naive patients with MDD of relatively short illness duration, and to examine correlations between ALFF and clinical measures.

Methodology: This cross-sectional observational study was conducted at the University of Health Sciences, Lahore, from June 2024 to January 2025. Thirty first-episode, drug-naive patients with MDD of relatively short illness duration (mean = 14 weeks), along with 52 healthy controls (HCs), were scanned with rs-fMRI to obtain ALFF across the whole brain. Voxel-based analysis of ALFF maps was performed to compare MDD and HC groups using a two-sample *t*-test. Correlations between ALFF and symptom severity measured by the Hamilton Rating Scale for Depression (HAMD) score or illness duration were conducted using simple regression.

Results: Compared with HC, patients with MDD had increased ALFF in the dorsal anterior cingulate cortex (dACC) and vermal sub-regions V3 of the cerebellum; no areas of significantly decreased ALFF were found. There was no correlation between the elevated ALFF value and clinical parameters; only the ALFF value in the right dorsolateral prefrontal cortex (DLPFC) was found to correlate negatively with the HAMD score in patients with MDD.

Conclusions: Our study demonstrates that alterations of cerebral intrinsic activity may occur in the early course of MDD without interference from antidepressants. As these regions are crucial for the regulation of cognition, we speculate that these changes may subserve the disturbances of cognitive function in early MDD.

## Introduction

Major depressive disorder (MDD), a common mental health condition, is characterized by low mood, anhedonia, lack of interest, and low energy [1]. Given its highly disabling nature and the high lifetime risk of suicide [2], better advances in MDD treatment require a deeper understanding of its neurobiological pathophysiology. Resting-state fMRI (rs-fMRI), which does not require designated task paradigms, is a noninvasive tool capable of measuring spontaneous brain activity, offering valuable insights into psychiatric disorders [3,4]. Research studies employ amplitude of low-frequency fluctuation (ALFF) as an rs-fMRI index because it detects spontaneous blood oxygenation level-dependent (BOLD) signal fluctuations, allowing measurement of regional intrinsic brain activity [5]. ALFF has demonstrated high test-retest reliability and temporal stability [6], making it a robust tool for investigating intrinsic brain activity disturbances in psychiatric conditions such as depression and schizophrenia [7], autism spectrum disorder [8], and attention-deficit/hyperactivity disorder [5].

The literature demonstrates that MDD affects ALFF across various brain regions, including the anterior cingulate cortex (ACC) [9-12], parahippocampal gyrus, ventromedial frontal gyrus, and putamen [12]. The variability in study results may stem from disease heterogeneity, as well as medication status and illness duration [13]. Patients treated without antidepressant medications demonstrated decreased ALFF levels in the bilateral orbitofrontal cortex, while showing increased ALFF levels in the bilateral temporal cortex [14]. Researchers have suggested that patients' medication status may explain the differences observed between studies using a cross-sectional design. However, an increasing number of longitudinal studies now demonstrate that MDD-related abnormal brain activation patterns in specific regions can be normalized through antidepressant treatment [15]. Antidepressant treatment restores normal functional activity in the DLPFC during cognitive testing in patients with MDD. Post-treatment with escitalopram led to increases in fALFF in the DLPFC, while depressed patients still exhibited decreased fALFF signals compared to healthy controls at baseline [16].

The objective of this study was to investigate alterations in intrinsic brain activity using voxel-wise ALFF analysis in first-episode, drug-naive patients with MDD, and to assess the correlation between these alterations and depression severity, as measured by Hamilton Rating Scale for Depression (HAMD) scores.

## Materials and methods

This cross-sectional observational study was conducted at the University of Health Sciences, Lahore, from June 2024 to January 2025. This study was approved by the local ethical committee, and written informed consent was obtained from all participants. Thirty patients with first-episode, drug-naive MDD and fifty-two healthy controls (HCs) participated in this study. The patients were part of a large cohort study on major depression in the Pakistani population. All patients were diagnosed using the Structured Clinical Interview for DSM-IV Axis I Disorders (SCID), with diagnoses confirmed by two experienced psychiatrists. The severity of depression was quantified using the 17-item HAMD. The inclusion criterion was a HAMD total score greater than 18 on the day of magnetic resonance imaging (MRI) scanning. The exclusion criteria for patients were as follows: psychiatric disorders based on DSM-IV other than MDD, any history of major illness, previous psychiatric therapy, cardiovascular disease, age under 18 or over 60 years, use of vasoactive medications, and alcohol or drug abuse. None of the patients had received antidepressant treatment before enrolment. HCs were recruited from the same sociodemographic environment through poster advertisements. Controls were screened using the non-patient edition of the SCID. They were required to be free from psychiatric or neurological illness and have no family history of psychiatric disorders. All participants were right-handed.

MRI data acquisition

All participants underwent scanning using a 3.0T MR imaging system (EXCITE, GE Signa, Milwaukee) with an eight-channel phased-array head coil. rs-fMRI data, sensitized to changes in BOLD signal levels, were obtained using a gradient-echo echo-planar imaging (EPI) sequence with the following parameters: repetition time = 2000 ms, echo time = 30 ms, flip angle = 90°, slice thickness = 5 mm, intersection gaps = 0, matrix size = 64 × 64, field of view = 240 × 240 mm², and voxel size = 3.75 × 3.75 × 5 mm³. Each brain volume comprised 30 axial slices. During the rs-fMRI scan, each participant used soft earplugs and foam cushions to decrease noise jamming and head motion. Participants were instructed to relax with their eyes closed, but to avoid falling asleep and engaging in directed or systematic thought, which was confirmed by the subjects immediately after scanning. Data were excluded from the study if head movement exceeded 1.5 mm or 1.5°.

Data preprocessing

rs-fMRI data preprocessing was carried out using the Data Processing Assistant for Resting-State Functional MR Imaging software. For each participant, the first five volumes were discarded to allow for steady-state stabilization. The remaining images were section-time corrected, realigned to the middle volume, and unwrapped to correct for susceptibility-by-movement interaction. Realigned images were spatially normalized to the Montreal Neurological Institute (MNI) EPI template.

Amplitude of low-frequency fluctuation

ALFF was calculated using DPARSF software with a procedure similar to that employed in our earlier study [17]. After band-pass filtering (0.01-0.08 Hz) [18] and removal of linear trends, the time series were transformed into the frequency domain using fast Fourier transform (FFT) (taper percentage = 0; FFT length = shortest). The power spectrum was obtained and transformed to the square root and then averaged across 0.01-0.08 Hz at each voxel. The averaged square root was taken as the ALFF. For the standardization procedure, the ALFF of each voxel was divided by the global mean ALFF value (similar to approaches used in positron emission tomography studies [19]). Finally, spatial smoothing was applied using an 8-mm full-width-at-half-maximum Gaussian kernel.

Statistical analysis

Demographic and clinical differences between patients with MDD and HCs were tested using the independent-samples t-test for continuous variables and the chi-square test for categorical variables, with analysis conducted using SPSS 19.0 (IBM Corp., Armonk, NY). The value of α was set at *P* < 0.05.

## Results

The mean age of participants in the MDD group was 36.27 years (SD = 12.05), while the HC group had a comparable mean age of 35.38 years (SD = 12.31), with no significant difference observed (*P* = 0.754). The proportion of male participants was slightly higher in the HC group (23, 44.2%) compared to the MDD group (8, 26.7%), though this difference was not statistically significant (*P* = 0.114). The MDD group had a mean of 12.33 years of education and an average illness duration of 14.13 weeks (SD = 16.39). The mean HAMD score for the MDD group was 24.20 (SD = 5.08), consistent with moderate-to-severe depressive symptoms (Table 1).

**Table 1:** Demographic and clinical characteristics of all participants. A *P*-value of <0.05 was considered statistically significant. MDD, major depressive disorder; HC, healthy control; HAMD, Hamilton Rating Scale for Depression; SD, standard deviation

Variable	MDD (*n* = 30)	HC (*n* = 52)	*P*-value
Age, mean (SD) (years)	36.27 (12.05)	35.38 (12.31)	0.754
Gender, *n* (% male)	8 (26.7%)	23 (44.2%)	0.114
Education, mean (SD) (years)	12.33 (4.53)	14.21 (3.87)	0.032
Illness duration, mean (SD) (weeks)	14.13 (16.39)	-	-
HAMD score, mean (SD)	24.20 (5.08)	3.12 (1.97)	<0.001

**Figure 1:**
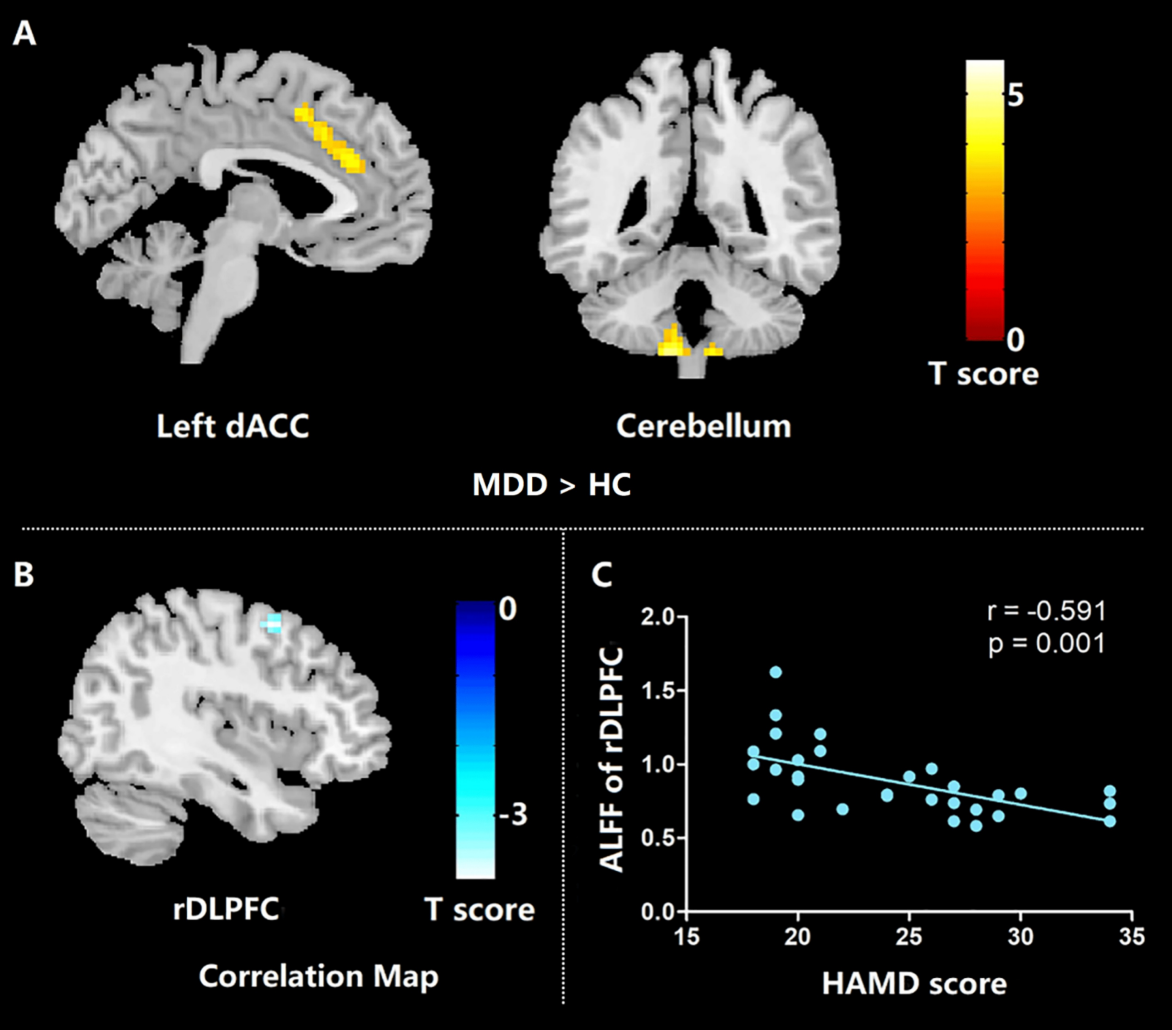
Results of ALFF differences between first-episode, drug-naive patients with MDD and healthy controls (A), and correlation maps between ALFF and HAMD scores for patients with MDD (B, C). MDD, major depressive disorder; HC, healthy controls; ALFF, amplitude of low-frequency fluctuation; HAMD, Hamilton Rating Scale for Depression score; rDLPFC, right dorsolateral prefrontal cortex

The most prominent differences were observed in the cerebellum, with a large cluster (*n* = 158) showing peak activation at MNI coordinates *x* = -12, *y* = -48, *z* = -60 (*T* = 5.71), along with additional significant foci at *x* = 12, *y* = -51, *z* = -60 (*T* = 4.95) and *x* = 9, *y* = -42, *z* = -57 (*T* = 4.57). Increased activity was also detected in the left dorsal anterior cingulate cortex (dACC), with a cluster size of 89 and peak activation at *x* = -6, *y* = 36, *z* = 21 (*T* = 4.31), alongside adjacent coordinates demonstrating elevated *T* values (Table 2; Figure 1).

**Table 2:** Region-specific differences in ALFF between first-episode, drug-naive patients with MDD and HCs. *T-statistical values of peak voxels showing ALFF differences between first-episode, drug-naive patients with MDD and HCs. The statistical threshold was set at *P* < 0.001 at the individual voxel level, corrected for multiple comparisons using familywise error correction at the cluster level (*P* < 0.05). MDD, major depressive disorder; HC, healthy control; ALFF, amplitude of low-frequency fluctuations; dACC, dorsal anterior cingulate cortex

Group comparison	Brain region	Cluster size	x	y	z	*T*-value	*P*-value
MDD > HC	Cerebellum	158	-12	-48	-60	5.71*	<0.001
			12	-51	-60	4.95	<0.001
			9	-42	-57	4.57	<0.001
	Left dACC	89	-6	36	21	4.31*	<0.001
			-6	21	36	3.83	<0.001
			-9	24	27	3.77	<0.001

## Discussion

In this study, drug-naive MDD patients with a first episode, relatively short illness duration, and resting cerebral intrinsic activity were examined. The findings showed that these patients with MDD had elevated ALFF in the dACC and vermal sub-region V3 of the cerebellum. This suggests that, in the early stages of MDD, cerebral intrinsic activity was disrupted without interference from medication. In addition, negative correlations were found between the HAMD score and the ALFF in the right DLPFC, indicating that the cerebral intrinsic activity of the right DLPFC may serve as a measure of depression severity in patients with MDD. Our study provided new insights into the neurobiology of depression's early stages [20]. According to cytoarchitecture and functional differences, the ACC can be divided into an *affective subdivision* that includes the rostral ACC (rACC) and the subgenual ACC (sgACC), and a *cognitive subdivision* that includes the dorsal ACC (dACC). The dACC constitutes an important node in a cognitive control network, which engages in complex cognitive tasks such as working memory, decision-making, and conflict resolution [21]. A recent meta-analysis of the neuroimaging literature confirmed that the dACC is involved in tasks that require cognitive control [22]. Meta-analysis of cognitive deficits showed that reduced executive function was a characteristic marker of first-episode MDD. For those patients with reduced executive function, the function of dACC may need to be enhanced to perform normal cognitive tasks such as decision-making or conflict resolution [23]. In the present study, we found increased ALFF in the dACC of early-course, untreated patients with MDD. Therefore, we hypothesize that the increased intrinsic dACC activity in first-episode, drug-naive patients may represent a novel preventative or delayed mechanism for their decline in executive functions. However, this conclusion requires further verification through future animal experiments.

When compared to HCs, our study found that first-episode, drug-naive patients with MDD had higher ALFF activity in the dACC. In line with this, Liu et al. also found increased ALFF activity in the dACC in drug-naive patients with MDD. However, a previous meta-analysis of eight ALFF studies demonstrates that ACC activity increased only in the treated patients but not in drug-naive patients with MDD [24]. That meta-analysis included five original studies on drug-naive patients, and four out of five of these drug-naive studies failed to find ALFF activation differences in the ACC between patients with MDD and HCs. This discrepancy may be due to the smaller sample size [14] and lower field strength of the MR scanner [11] in those studies compared to ours. ALFF in the vermal sub-region V3 of the cerebellum was also significantly higher in patients with MDD than in controls. Functional abnormalities in the cerebellum are a common manifestation of MDD.

There were several limitations in our study. Future studies should simultaneously record the cardiac and respiratory rates to solve these potential confounding variables. Second, because our study is cross-sectional, longitudinal studies should investigate whether these abnormal ALFF values change dynamically after therapy. Third, the results of our study cannot be generalized to patients with MDD who have a long illness duration or have previously been treated with other medications, due to the nature of our cohort of first-episode, drug-naive patients and the relatively small sample size. Nevertheless, our findings may provide important new insights into the early neurobiology of depression. Further studies with larger sample sizes are needed to confirm our results.

## Conclusions

In conclusion, our study identified the dACC and vermal sub-region V3 of the cerebellum as being closely associated with drug-naive patients with MDD during their first episode of depression, suggesting a disturbance of cerebral intrinsic activity in the early course of MDD, without the interference of medication. As these two regions play an important role in cognitive function, it is speculated that the early increased cerebral intrinsic activity in these regions may prevent or delay the progression of cognitive impairment in patients with MDD at an early stage. This could have significant clinical implications for early screening and appropriate treatment of these patients. In addition, our study indicated that the right DLPFC may be clinically valuable in evaluating depression severity in first-episode, drug-naive patients with MDD, providing new insights into the neurobiology of the early course of depression.
